# The role of nucleus accumbens Fos expression in sex-dependent cocaine-induced locomotion, cocaine self-administration, and primed cocaine seeking in rats

**DOI:** 10.1016/j.bbr.2026.116055

**Published:** 2026-01-22

**Authors:** Alixandria T. Mascarin, Srinivasu Kallakuri, Justin L. Carthage, Ali Gheidi, Alana C. Conti, Shane A. Perrine

**Affiliations:** a Translational Neuroscience Program Department of Psychiatry and Behavioral Neurosciences, School of Medicine, Wayne State University, 6135 Woodward Ave, Detroit, MI 48202, United States; b Department of Psychology, Wayne State University, 5047 Gullen Mall, Detroit, MI 48202, United States; c Department of Biomedical Sciences, School of Medicine, Mercer University, 1550 College St, Macon, GA 31207, United States; d John D. Dingell Veteran Affairs Medical Center, 4646 John R St, Detroit, MI 48201, United States

**Keywords:** Locomotor response, Cocaine self-administration, Cocaine seeking, Fos, Nucleus accumbens, Neuronal ensembles, Sex differences

## Abstract

Individuals using cocaine, particularly those with Cocaine Use Disorder, experience long-lasting neurobiological alterations that contribute to high rates of relapse and increased morbidity and mortality. Rodent models suggest that neuronal activation, as represented by Fos expression, in the nucleus accumbens (NAc) is crucial for cocaine-related behaviors. However, the role of biological sex in NAc activation in these behaviors remains unclear. Therefore, the present study examined the impact of sex on cocaine-induced locomotor activity (LMA), cocaine self-administration, cocaine seeking, and associated NAc Fos expression. We hypothesized that, relative to males, female rats would display heightened behavioral responses in the tested models and greater numbers of Fos+ cells in the NAc, and that Fos expression would correlate with the outcome measures of the assessed behaviors (i.e., locomotor activity and cocaine seeking). In this study, females displayed greater cocaine-induced locomotor activity, cocaine self-administration, and cocaine seeking than males. However, neither sex nor cocaine treatment impacted NAc Fos expression in the LMA study, and NAc Fos levels did not correlate with LMA in either sex. Following cocaine seeking, NAc Fos expression was not sex-dependent, though it correlated with cocaine seeking in males, but not in females. Taken together, these results suggest that the number of Fos+ cells in the NAc do not underlie sex differences in cocaine use or relapse-like behaviors. Future work should characterize the proteomic or electrophysiologic profiles in specific cell types of Fos+ cells in the NAc following cocaine use to determine how these behaviors differ by sex.

## 1. Introduction

Cocaine users experience high relapse rates, morbidity, and mortality [1] driven in part by lasting neurobiological and behavioral changes induced by chronic cocaine use [2]. Animal models of cocaine activity, use and relapse, such as behavioral sensitization, self-administration, and cocaine seeking, have facilitated our understanding of the neurobiological mechanisms underlying relapse. Evidence from these and other models suggests that the nucleus accumbens (NAc) has a defined role in motivated behavior related to substance use [2–4] and that neuronal activation in the NAc, indicated by cellular expression of the activity-dependent protein Fos, results from and is critical for expression of these cocaine-related behaviors. For example, cocaine-induced context-specific sensitization is associated with increased NAc Fos expression [5–7]. Fos expression in the NAc is sensitized in response to repeated cocaine [8,9] and NAc Fos+ neurons are necessary for cocaine-seeking behavior [10,11]

Importantly, the above studies investigate Fos expression in the NAc only in males. Sex differences in cocaine use have been reliably demonstrated both clinically and in animal models [12–16]. Women exhibit greater reactivity to cocaine-associated cues [17], experience greater cravings for cocaine during abstinence [18], and suffer from higher relapse rates in treatment compared to men [19]. Similarly, female rats show greater cocaine-induced behavioral sensitization [20], acquire cocaine self-administration faster [21], and display heightened cocaine-seeking behavior [22]. Despite these known sex differences in cocaine use across species, Fos expression in the NAc underlying cocaine-related behaviors have largely been studied only in male rats [10,11]. Research examining this mechanism in both sexes remains unclear. Zhou et al. report greater Fos expression in the NAc shell, but not NAc core, in females than males following cue-induced cocaine seeking [23], but this limited research requires further exploration.

Therefore, this study examined Fos expression in the NAc in female and male rats using two models of cocaine use: repeated cocaine-induced locomotor activity (LMA; Experiment 1) and cocaine self-administration and cocaine seeking (Experiment 2). We hypothesized that females would display greater cocaine-induced sensitization of LMA, cocaine self-administration, and primed cocaine seeking than males, along with more NAc Fos+ cells. Finally, we hypothesized that LMA (Experiment 1) and cocaine seeking (Experiment 2) would correlate with the number of Fos+ neurons in the NAc in both sexes.

## 2. Methods

### 2.1. Animals

All procedures were approved by the Wayne State University Institutional Animal Care and Use Committee and followed the guidelines in the Guide for the Care and Use of Laboratory Animals [24]. Rats were kept on a reversed 12 h light-dark cycle (lights off 6 AM) with standard environmental and housing conditions. Animals were pair-housed by sex with ad libitum access to food (LabDiet) and water for the duration of the study. Males and females were run in separate cohorts to minimize potential confounds, and all testing environments were cleaned thoroughly between animals.

Experiment 1 included 48 (24 male, 24 female) adult Sprague Dawley rats (Charles River Laboratories, Portage, MI). Animals were allowed to habituate upon arrival for six days before the experimental paradigm began. Due to technical issues during immunofluorescence, six animals were excluded from Fos analyses. Experiment 2 included 32 (16 female) Wistar rats bred at Wayne State University. These rats were heterozygous for the Fos-LacZ transgene (Wistar-Tg(Fos-LacZ)1Ottc) [25], but the transgene was not implemented in this study. As 13 of the 32 rats failed to meet acquisition criteria and were subsequently excluded, behavioral analyses were comprised of 19 rats (12 female, 7 male). Of these 19 rats, 3 female subjects were included in behavioral analyses, but excluded from Fos analyses, due to technical issues during immunofluorescence procedures.

### 2.2. Intravenous catheter implantation surgery

Catheter implantation occurred on or about post-natal day 70 when females weighed 150–250 g and males weighed 300–400 g, as previously described [26,27]. Briefly, rats were anesthetized with 5 % inhaled isoflurane in oxygen, and polyurethane catheters (Instech Laboratories; Plymouth Meeting, PA) were implanted into the right jugular vein using sterile, aseptic techniques. Catheters were connected to a vascular access button (Instech Laboratories) positioned posterior to the shoulder blades. Carprofen (5 mg/kg) was dissolved in sterile saline and administered intraperitoneally before surgery and once daily for 3 days after surgery to relieve pain. Seven to ten days after surgery rats began cocaine self-administration. Throughout the paradigm, catheters were flushed weekly with 0.1 mL of 20 USP sterile heparinized saline flush solution. Resistance during flushing indicated loss of patency, but animals were not excluded on this basis alone. Rather, acquisition criteria were defined as > 50 % active lever pressing in the last 3 acquisition sessions.

### 2.3. Drugs

[−]Cocaine HCl was obtained from the National Institute on Drug Abuse Drug Supply Program. Cocaine was dissolved in sterile saline (0.9 % NaCl) at concentrations of 10 mg/mL and 4 mg/mL for Experiments 1 and 2, respectively. In Experiment 1, cocaine was injected intraperitoneally at a dose of 10 mg/kg at 1 mL/kg volume. In Experiment 2, cocaine was infused intravenously at a dose of 0.5 mg/kg/infusion on a fixed-ratio 1 (FR-1) schedule of reinforcement with 20-second timeouts between drug-available periods. Cocaine dose in Experiment 2 was maintained by adjusting infusion delivery duration on the fixed-rate infusion pump.

### 2.4. Apparatus

#### 2.4.1. Open field

Locomotor activity (LMA) assessments occurred in an open-topped black Plexiglas box measuring 80 × 80 × 36 cm (Form Tech Plastics, Oak Park, MI), and behavior was video recorded from overhead for 15 min. These recordings were analyzed using Ethovision XT 11 (Noldus Information Technology, Wageningen, The Netherlands) to determine the total distance traveled.

#### 2.4.2. Self-administration chambers

Rats underwent cocaine-taking and seeking sessions individually in Med Associates self-administration chambers (53.34 ×34.93 ×27.31 cm). The chambers were housed in isolation cubicles and equipped with two levers (active and inactive), lever-associated lights, and a red house light. Rats were connected to a magnetic tether system to allow free movement around the chamber, which included a single-channel vascular access button tether kit (Med Associates; Fairfax, VT) magnetically attached to the vascular access button (Instech Labs) surgically implanted in the rat. Chambers were equipped with a fixed-rate infusion delivery pump that was connected via polyurethane tubing to the tether system (Med Associates).

### 2.5. Experimental paradigms

#### 2.5.1. Experiment 1: repeated cocaine-induced LMA

**Fig. 1.**
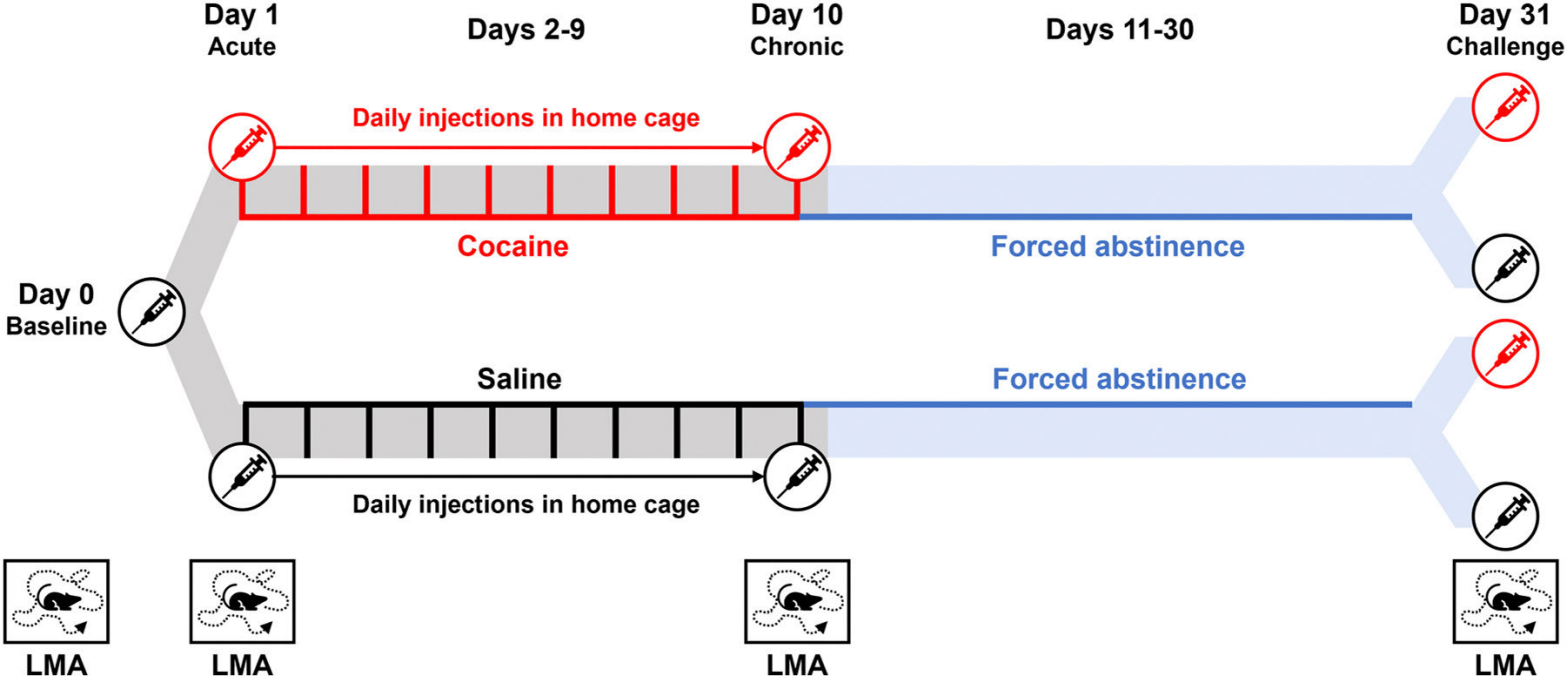
Timeline of Experiment 1 – Behavioral Sensitization. Red and black lines and/or syringes represent cocaine and saline injections, respectively. Blue lines represent the abstinence period in the home cage. Boxes at the bottom of the image indicate days of LMA assessment.

As shown in Fig. 1, Experiment 1 assessed whether repeated cocaine treatmentsinduced behavioral sensitization of LMA over 31 days. On day 0, all animals received a saline injection, and baseline LMA was recorded. On day 1, animals were pseudorandomly assigned to either cocaine or saline treatment and LMA was assessed following acute cocaine or saline administration. From days 2–9, animals were given single daily injections of either saline or cocaine in the home cage with no LMA testing. On day 10, animals were given an injection of either saline or cocaine and LMA was assessed. Animals were then subjected to forced abstinence in the home cage on days 11–30. On day 31, animals were subdivided to create a 2 × 2 design and receive cocaine or saline challenge injections and LMA was assessed.

#### 2.5.2. Experiment 2: cocaine self-administration and cocaine seeking

**Fig. 2.**
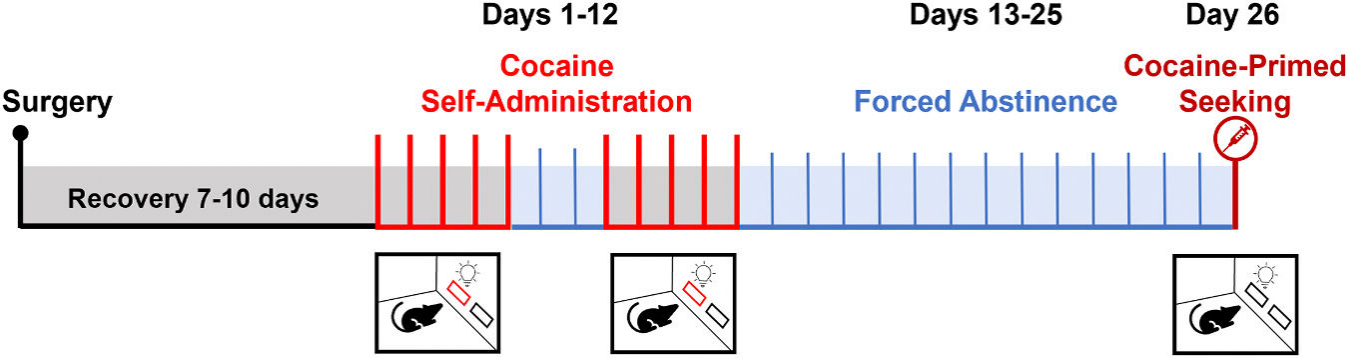
Timeline of Experiment 2 – Self-Administration. Bright red lines indicate days of self-administration sessions. Blue lines represent forced abstinence. The dark red line depicts the cocaine-seeking session. Boxes on the lower portion of the figure indicate days where behavioral testing occurred. Red lever in box indicates lever presses were reinforced with cocaine (i.e., self-administration), whereas both black levers indicated no reinforcement of operand (i.e., cocaine seeking).

Experiment 2 consisted of three phases, shown in Fig. 2: [1] acquisition of cocaine self-administration for ten sessions over days 1–12, [2] forced abstinence on days 13–25, and [3] a 30-minute cocaine-primed seeking session on day 26. Rats were trained to self-administer cocaine for 3 h/session for 10 sessions. Self-administration sessions occurred over 12 days with a 2-day forced abstinence period in the home cage between sessions 5 and 6. During self-administration sessions, both levers were extended, and the light associated with the active lever was turned on. Active lever presses initiated the infusion pump to deliver cocaine and induced a 20-second timeout period, wherein both levers were retracted, the lever light was inactivated, and the house light was turned on. Inactive lever presses had no programmed consequence but were recorded. Animals were required to meet acquisition criteria (>50 % of total lever presses on active lever in the last 3 acquisition sessions). Thirteen animals that failed to reach these acquisition criteria were excluded from the study. During forced abstinence periods, the rats were confined to their home cage without access to cocaine or the self-administration chamber. Immediately before the cocaine-seeking session, all rats were given a priming injection of cocaine (10 mg/kg, intraperitoneal) and placed in the chamber. Cocaine-primed seeking sessions (30 min) were recorded and conducted under the same conditions as the acquisition sessions (3 h), except that active lever presses did not deliver cocaine. Following the seeking session, animals were returned to the home cage in a dark, quiet environment until euthanasia and tissue collection.

### 2.6. Tissue preparation and immunofluorescence for Fos

At ninety minutes from the start of the final behavioral session, a time period that allows for maximal Fos expression [28], rats were deeply anesthetized with 5 % isoflurane in oxygen and perfused with 200 mL of phosphate-buffered saline (PBS) followed by 200 mL of 4 % paraformaldehyde (PFA) in deionized water. The brains were removed and post-fixed in PFA for 3 days at room temperature, transferred to 15 % sucrose in 1XPBS solution at 4°C for 3 days, then 30 % sucrose in 1XPBS at 4°C for 3 days. Brains were then frozen in powdered dry ice and kept at −80°C until sectioning. Coronal sections containing the NAc were cut at 40 μm thick sections between bregma + 2.0 and + 1.6 mm using a cryostat [29]. Three free-floating sections per animal were selected at approximately bregma + 1.6, + 1.8, and + 2.0 mm. Sections were washed three times in 1XPBS, blocked with 2 % normal donkey serum (NDS) in 1XPBS with 0.1 % Triton X-100 (1XPBS-Tx), and incubated for 2 days at 4°C with an anti-Fos antibody in blocking buffer (1:250 dilution, Cell Signaling, catalog # 74620S). The next day, sections were washed with 1XPBS and incubated for 2 h in Alexa 555 anti-mouse secondary antibody (1:500 dilution, Abcam, catalog # 150106) in 1XPBS-Tx and 2 % NDS at room temperature. After washing in 1XPBS, sections were mounted onto slides and coverslipped using a Fluoroshield DAPI-containing mounting medium (Millipore Sigma, catalog # DUO82040).

### 2.7. Microscopy and image analysis

Fluorescent images were obtained using a Leica THUNDER Imager Tissue microscope at 20x magnification. Templates for measuring immunofluorescence within the regions of interest (NAc core, NAc shell, and NAc combined [core and shell], and for a regional control, primary somatosensory cortex [pSSC] in Experiment 2) were constructed from rat brain atlas traces of coronal brain slices [29] and applied to all images in FIJI imaging software. Two independent, blinded raters counted co-localized DAPI and Fos expression in the NAc from both hemispheres of each of the prepared slices for a total of 6 counts per animal, except in cases of tissue damage that prevented analysis. Rater counts were averaged to quantify Fos expression in the NAc core and shell in Experiment 1 and NAc core, NAc shell, and pSSC in Experiment 2, and NAc combined counts were calculated by summing NAc core and shell counts. Inter-rater reliability for these counts was determined via Pearson correlation (R^2^ = 0.61 in Experiment 1; R^2^ = 0.86 in Experiment 2; data not shown).

### 2.8. Statistical analyses

For all analyses, significance was set at α = 0.05, and all statistics and graphs were produced with GraphPad Prism 10.

#### 2.8.1. Experiment 1

LMA across days 0, 1, and 10 was analyzed using a mixed model ANOVA, with sex [Male vs. Female] and treatment [Saline vs. Cocaine] as between-subject factors, day as the within-subject factor, and distance traveled (in cm) as the dependent variable. Tukey’s multiple comparisons tests were used for post-hoc analyses.

A three-way ANOVA was used to assess group differences on day 31, with sex [Male vs. Female], chronic treatment [Saline vs. Cocaine], and challenge injection [Saline vs. Cocaine] as between-subject factors, and distance traveled [30] as the dependent variable. Šídák’s multiple comparisons test was used for post-hoc analysis to compare groups differing by one factor.

Three-way ANOVAs tested group differences in Fos+ cells in the NAc Combined, NAc core, and NAc shell. These analyses included sex, chronic treatment, and challenge injection as between-subject factors and Fos+ cells as the dependent measure.

A three-way ANOVA was used to assess group differences between NAc core and shell Fos+ counts, with subregion, chronic treatment, and challenge injection as independent variables and the number of Fos+ cell counts as the dependent measure. Šídák’s multiple comparisons test was used for post-hoc analysis. Lastly, Pearson correlations were used to examine relationships between NAc Fos+ cell counts and LMA.

#### 2.8.2. Experiment 2

Cocaine-taking during acquisition was analyzed with a mixed-model ANOVA, using sex as the independent variable, session as the repeated measure, and the number of infusions or percent correct responding (number of active lever presses/total lever presses) as the dependent variable. Cocaine seeking after forced abstinence was analyzed with a two-way ANOVA with sex and lever (active vs. inactive) as the independent variables and lever presses as the dependent variable. Fisher’s LSD was used as a post-hoc analysis for the cocaine-seeking session. Paired *t*-tests were used to assess differences between NAc core and NAc shell and between NAc combined and pSSC Fos expression. Independent samples *t*-tests with Welch’s correction were used to determine differences between sexes on Fos expression in the NAc combined, NAc core, NAc shell, and pSSC. Spearman correlation and regression analyses assessed the relationship between Fos expression in the NAc combined and cocaine seeking.

## 3. Results

### 3.1. Experiment 1

#### 3.1.1. LMA

**Fig. 3.**
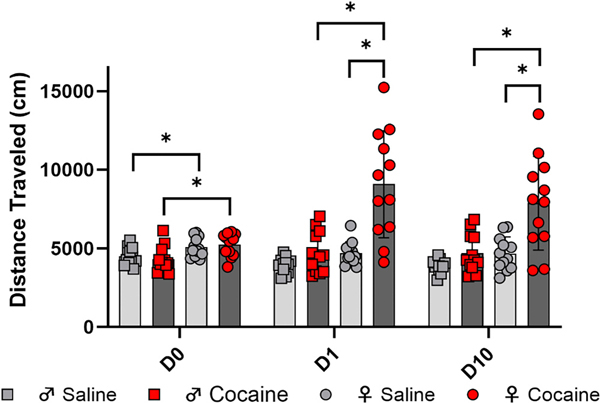
LMA on days 0 (baseline), 1 (acute), and 10 (chronic). ♂: females, shown in circles. ♀: males, shown in squares. Grey shapes with lighter bars represent saline-treated animals, and red shapes with darker bars indicate cocaine-treated animals. *=*p* < .05. Error bars represent standard error of the mean.

As shown in Fig. 3, the dose of cocaine used in this study did not induce behavioral sensitization in either sex but did induce hyperlocomotion in females. A mixed-model ANOVA revealed a significant three-way interaction of day, sex, and treatment [*F*(2, 88) = 5.077, *p* = 0.0082]; two-way interactions of sex and treatment [*F*(1, 44) = 10.63, *p* = 0.0022], day and treatment [*F*(2, 88) = 14.56, *p* < 0.0001], and day and sex [*F*(2, 88) = 6.467, *p* = 0.0024]; and main effects of day [*F*(1.88, 82.74) = 5.349, *p* = 0.0076], sex [*F*(1, 44) = 29.48, *p* < 0.0001], and treatment [*F*(1, 44) = 22.13, *p* < 0.0001] on LMA.

On day 0, baseline sex differences in LMA were evidenced by a main effect of sex in a two-way ANOVA [*F*(1, 44) = 14.88, *p* = 0.0004]. On day 1, a two-way ANOVA revealed a significant interaction of sex and treatment [*F*(1, 44) = 10.93, *p* = 0.0019], such that females treated with cocaine exhibited greater LMA than saline-treated females (*p* < 0.0001) and cocaine-treated males (*p* < 0.0001). On day 10, a significant interaction of sex and treatment [*F*(1, 44) = 5.882, *p* = 0.0195] suggested that cocaine-treated females continued to display greater LMA than their male counterparts (*p* < 0.0001) and saline-treated females (*p* < 0.0001).

Although we hypothesized an increase from day 1 to day 10 LMA in cocaine-treated animals (i.e., behavioral sensitization), post-hoc analysis did not reveal significant cocaine-induced differences between day 1 and 10 in either sex. The LMA of cocaine-treated males did not differ among days 0, 1, or 10. LMA of cocaine-treated females was significantly different on days 0 and 1 (*p* = 0.0041) and days 0 and 10 (*p* = 0.0138), but not days 1 and 10 (*p* = 0.4960).

On day 31, a three-way ANOVA revealed two-way interactions between sex and challenge exposure [*F*(1, 40) = 14.23, *p* = 0.0005] and sex and chronic treatment [*F*(1, 40) = 6.560, *p* = 0.0143], as well as main effects of sex [*F*(1, 40) = 57.18, *p* < 0.0001] and challenge exposure [*F*(1, 40) = 42.35, *p* < 0.0001), but no main effect of chronic treatment [*F*(1,40) = 1.955, *p* = 0.1697].

**Fig. 4.**
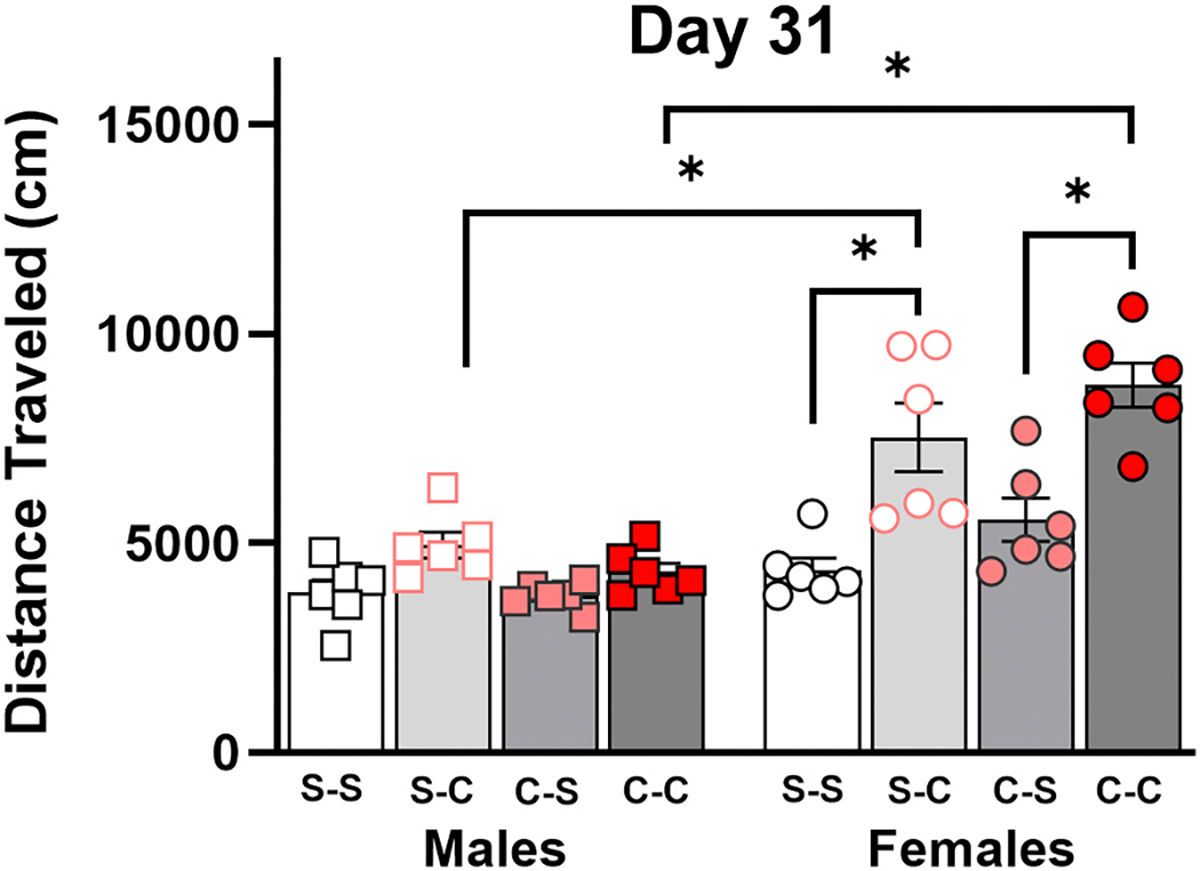
LMA on Day 31. The first letter in the group legend indicates chronic treatment (days 1–10), and the second letter represents challenge injection (day 31). *=*p* < .05. Error bars represent standard error of the mean.

As shown in Fig. 4, cocaine challenge injections increased LMA for (chronic) saline-treated and cocaine-treated females alike, but not for males. Females in the cocaine-cocaine group showed significantly higher LMA than both females in the cocaine-saline group (*p* < 0.0001) and males in the cocaine-cocaine group (*p* < 0.0001). Females in the saline-cocaine group displayed greater LMA than both females in the saline-saline group (*p* = 0.0001) and males in the saline-cocaine group (*p* = 0.0021). Although we hypothesized that a chronic cocaine treatment would sensitize the hyperlocomotor effect of the cocaine challenge, this was not supported by the data.

#### 3.1.2. Fos expression following LMA on Day 31

**Fig. 5.**
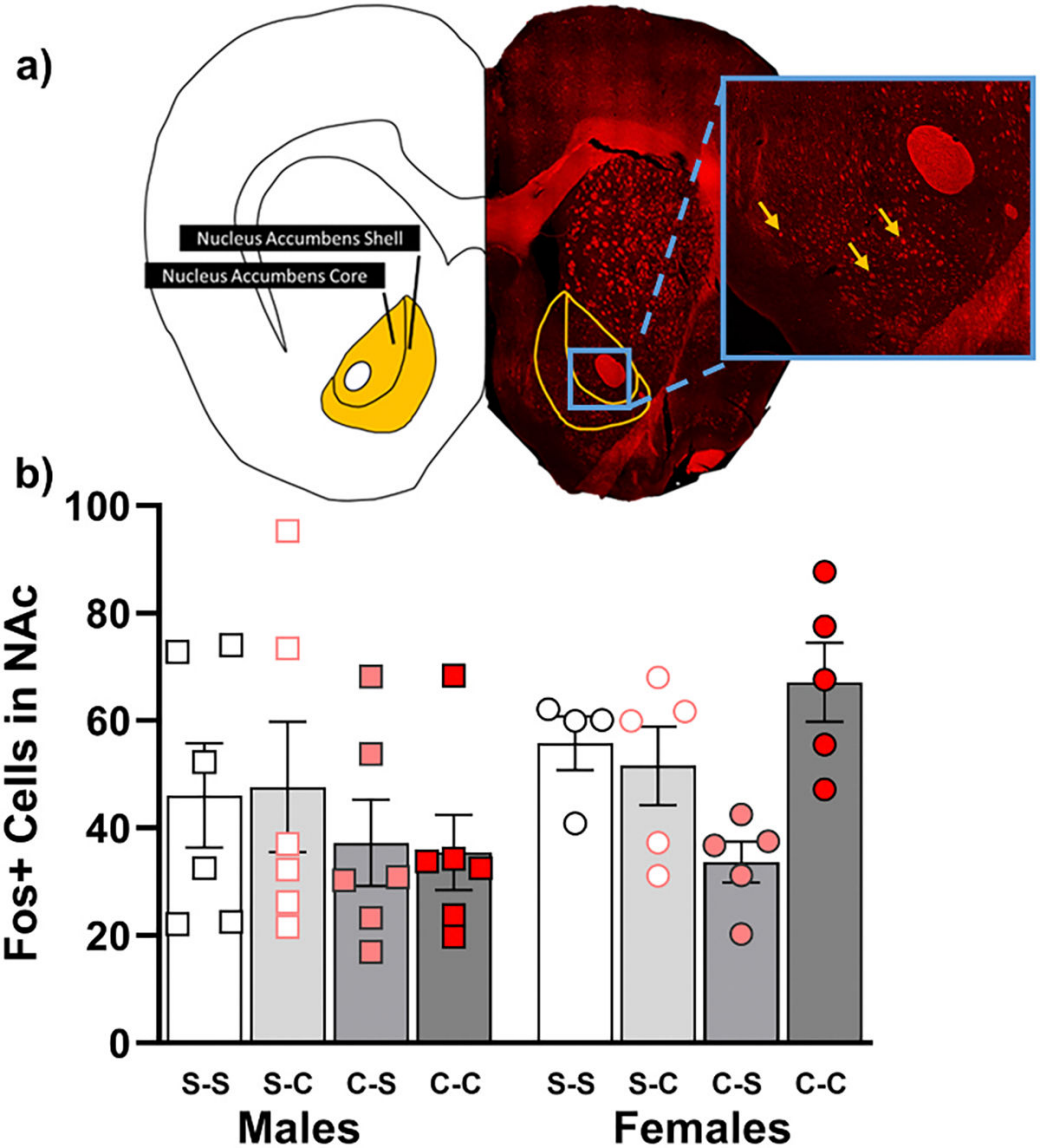
Fos expression following Day 31 LMA Assessment. a) Representative Fos image showing NAc subregions. Yellow arrows show examples of Fos+ cells. b) The first letter in the group legend indicates chronic treatment (days 1–10), and the second letter represents the challenge injection (day 31). Error bars represent the standard error of the mean.

Although we hypothesized that Fos expression would be greatest in the cocaine-cocaine group and heightened in females relative to males, a three-way ANOVA yielded no significant main or interaction effects of chronic treatment, challenge injection, or sex on Fos+ cells in the NAc core, NAc shell, or NAc combined (Fig. 5).

We did not hypothesize differences between subregion, but a three-way ANOVA revealed a main effect [*F*(1, 38)= 16.32, *p* = .0003], such that the NAc shell contained a greater number of Fos+ cells than the NAc core (data not shown). Post-hoc analyses revealed that this difference was significant only in the saline-saline group (*p* = .0407).

**Fig. 6.**
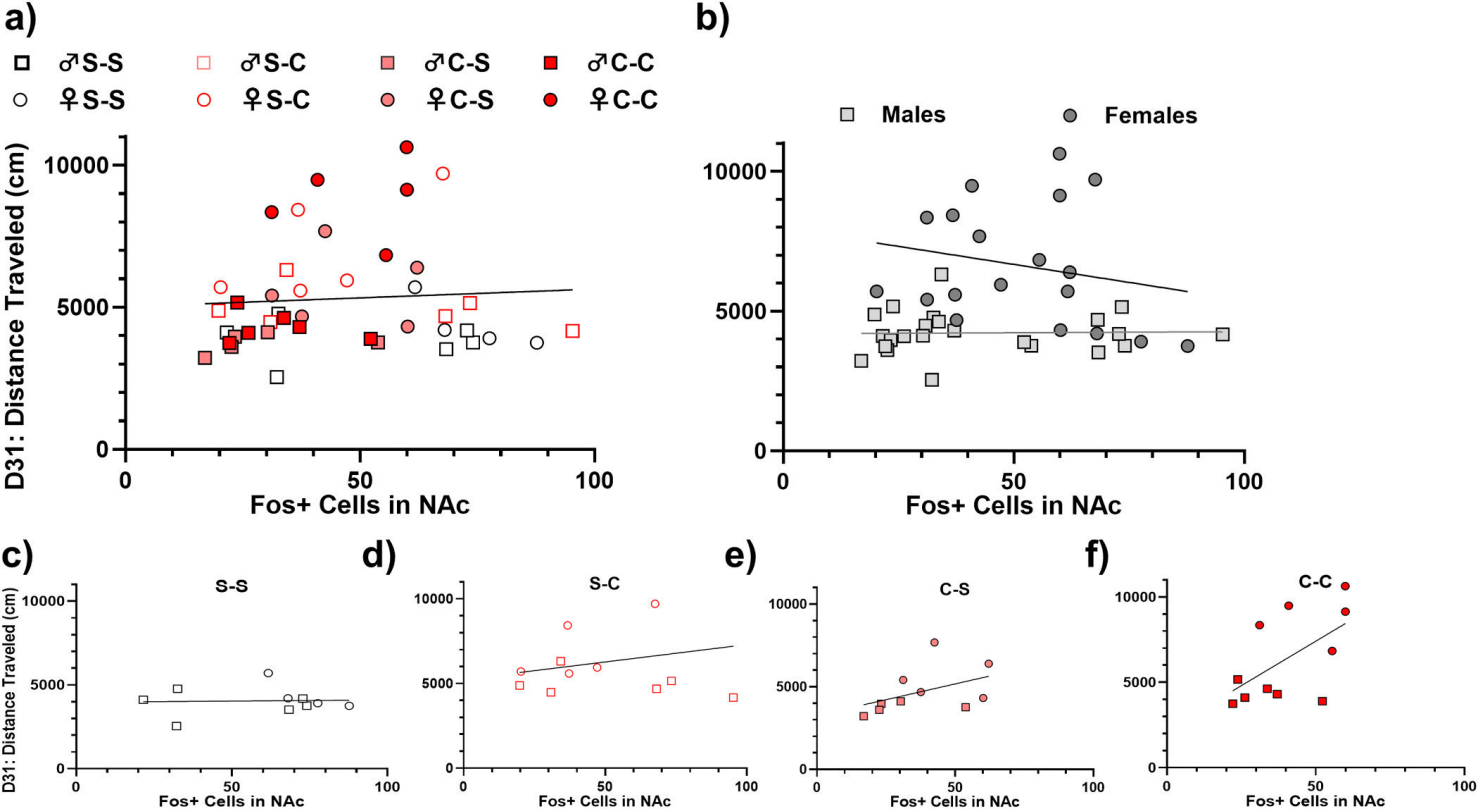
Relationship between NAc Fos expression and day 31 LMA. For all graphs, males are plotted in squares, and females are plotted in circles. The first letter in the group legend indicates chronic treatment (days 1–10) and the second letter represents the challenge injection (day 31). a) All groups, plotted individually. The first letter of legend represents chronic treatment, and the second letter represents challenge injection. b) Males and females, with treatment and challenge groups collapsed. c) Saline-saline males and females. d) Saline-cocaine males and females. e) Cocaine-saline males and females. f) cocaine-cocaine males and females.

We anticipated that NAc Fos expression would be correlated with Day 31 LMA. However, we found no evidence of a relationship. Whether collapsing all groups (R^2^=.0044, *p* = .6766; Fig. 6a) or separating by sex (males: [R^2^=0.0052, *p* = .9178]; females: [R^2^=.0449, *p* = .3836]; Fig. 6b) or by group (saline-saline: [R^2^=0.0014, *p* = .9183]; saline-cocaine: [R^2^=0.0031, *p* = .87]; cocaine-saline: [R^2^=0.1947, *p* = .2017]; cocaine-cocaine: [R^2^=0.3388, *p* = .0603]; Fig. 6c-f, respectively), no significant correlations were revealed. However, the cocaine-cocaine group showed a trending relationship (R^2^=.3388, *p* = 0.0603; Fig. 6f).

### 3.2. Experiment 2

#### 3.2.1. Cocaine-taking and cocaine-seeking behaviors

**Fig. 7.**
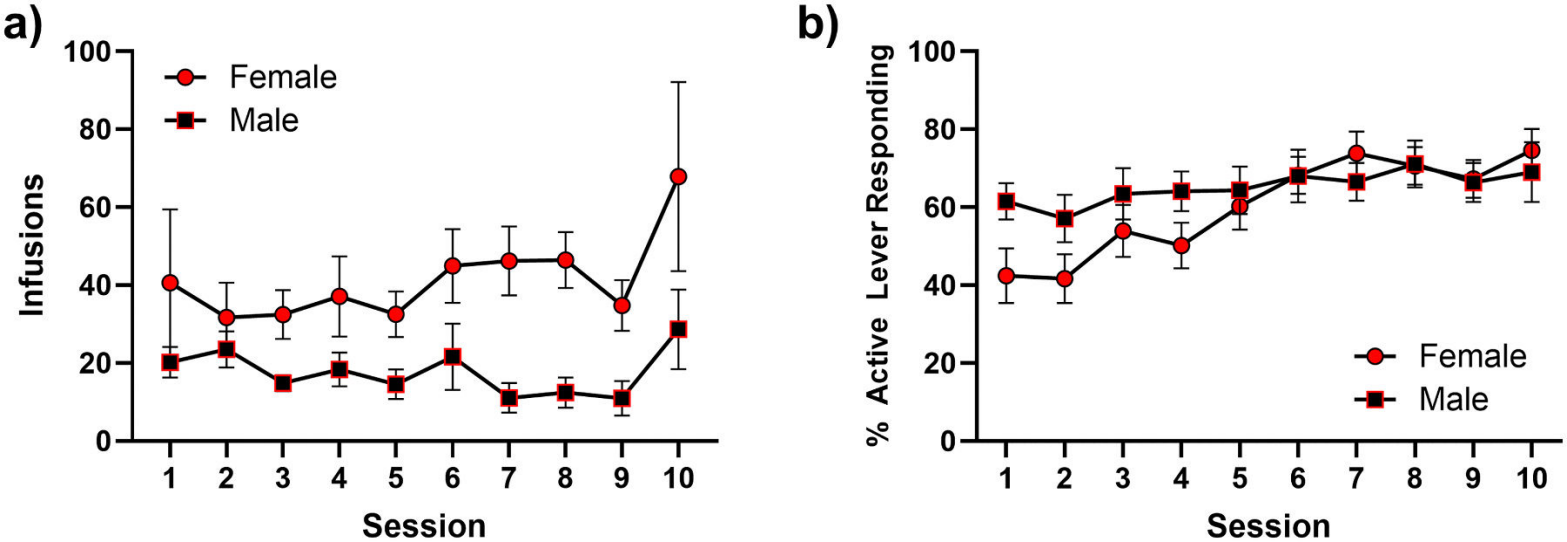
Acquisition of cocaine self-administration. a) Infusions of cocaine that males (black squares) and females (red circles) self-administered. b) % active lever responding, calculated as (active lever presses/total lever presses). Error bars represent the standard error of the mean.

**Fig. 8.**
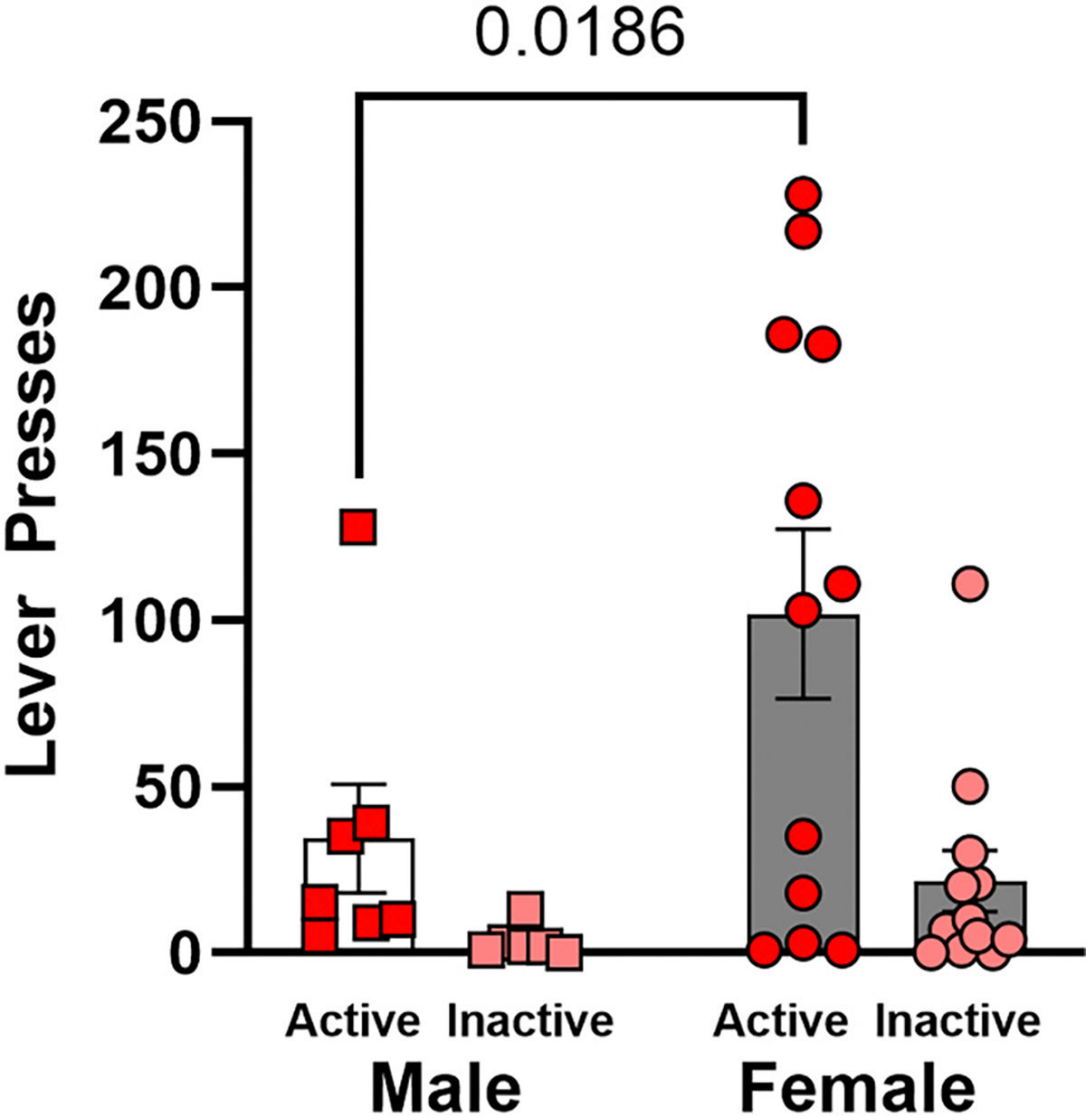
Active and inactive lever presses during the cocaine-seeking session. Error bars represent the standard error of the mean.

Animals improved on active lever responding, as indicated by a significant main effect of session, [F(3.312, 50.79) = 4.887; p = 0.0036; Fig. 7b], but females self-administered more infusions of cocaine across the 10 sessions [F(1, 17) = 6.181, p = 0.0236] (Fig. 7a). In the cocaine-seeking session, a two-way ANOVA revealed a main effect of lever [F (1, 16) = 8.673; p = 0.0095], but no significant main effect of sex [*F*(1, 17) = 4.367; *p* = 0.0520] or interaction of lever and sex [*F*(1, 16) = 1.671; *p* = 0.2145]. Hypothesis-driven, planned post-hoc comparisons revealed that females pressed the active lever significantly more than males [*t*(33) = 2.475; *p* = 0.0186], indicating that females exhibited greater cocaine-primed seeking behavior compared to male conspecifics (Fig. 8).

#### 3.2.2. Fos Expression following the cocaine-seeking session

**Fig. 9.**
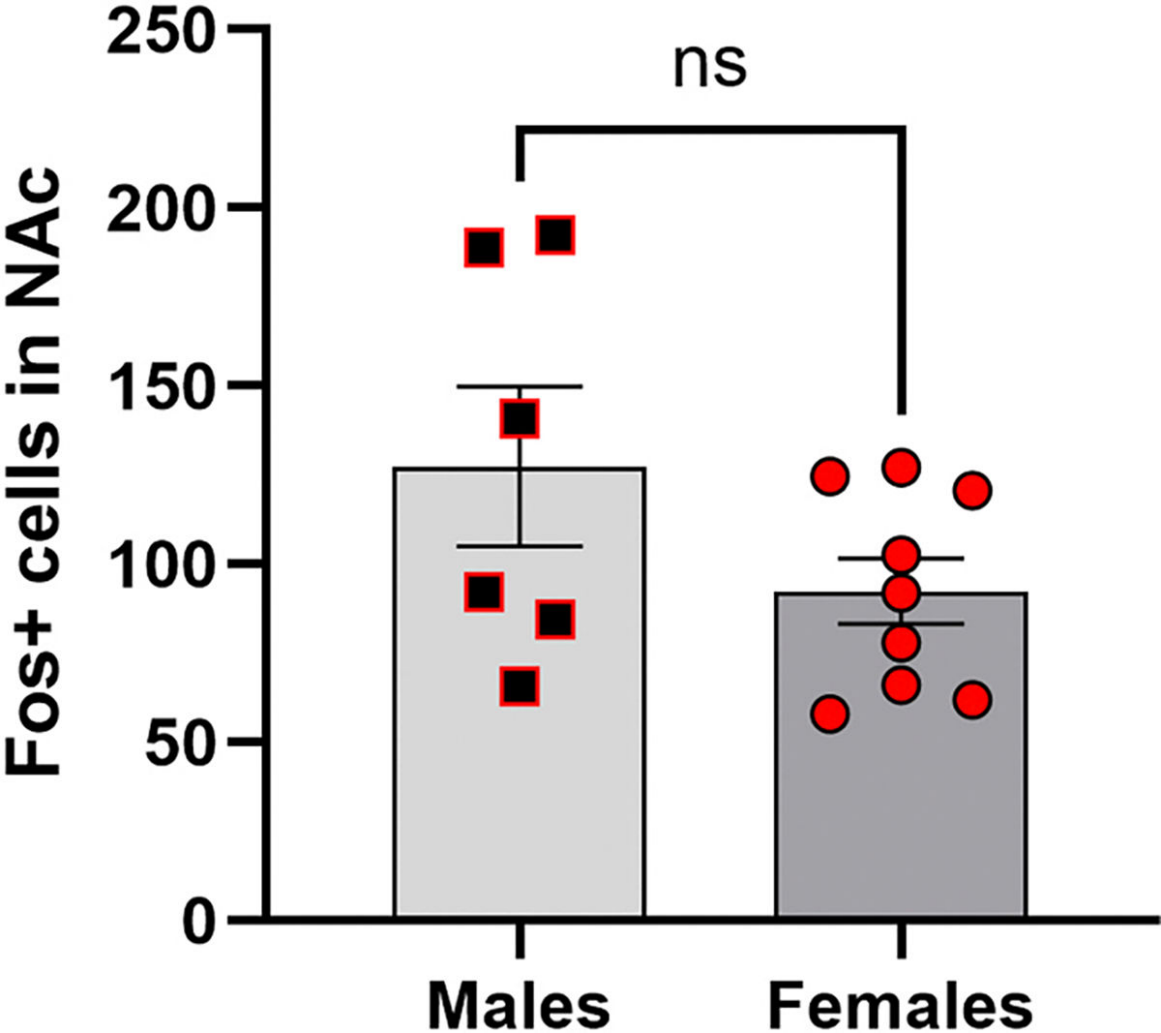
Fos expression following cocaine seeking. Error bars represent the standard error of the mean. NS: non-significant (*p* > .05).

Contrary to our hypothesis, females and males did not differ among the number of Fos+ cells in the NAc [*t*(6.732) = 1.445; *p* = 0.8008] (Fig. 9) or either subregion [core: *t*(6.958) = 1.365; *p* = 0.2148; shell: *t* (6.857) = 1.500; *p* = 0.1781] (data not shown) following cocaine-primed seeking behavior. NAc core and shell did not differ in the number of Fos+ cells (*p* = 0.8170; data not shown), and therefore, NAc combined cell counts were used for further analysis. To confirm whether Fos expression was specific to the NAc, we assessed the presence of Fos+ cells in the pSSC. The pSSC showed minimal Fos expression (mean = 5.635, SEM = 1.846) that was significantly different from the number of Fos+ cells in the NAc (*p* < 0.0001; data not shown) but not significantly different between sexes (*p* = 0.8126; data not shown).

**Fig. 10.**
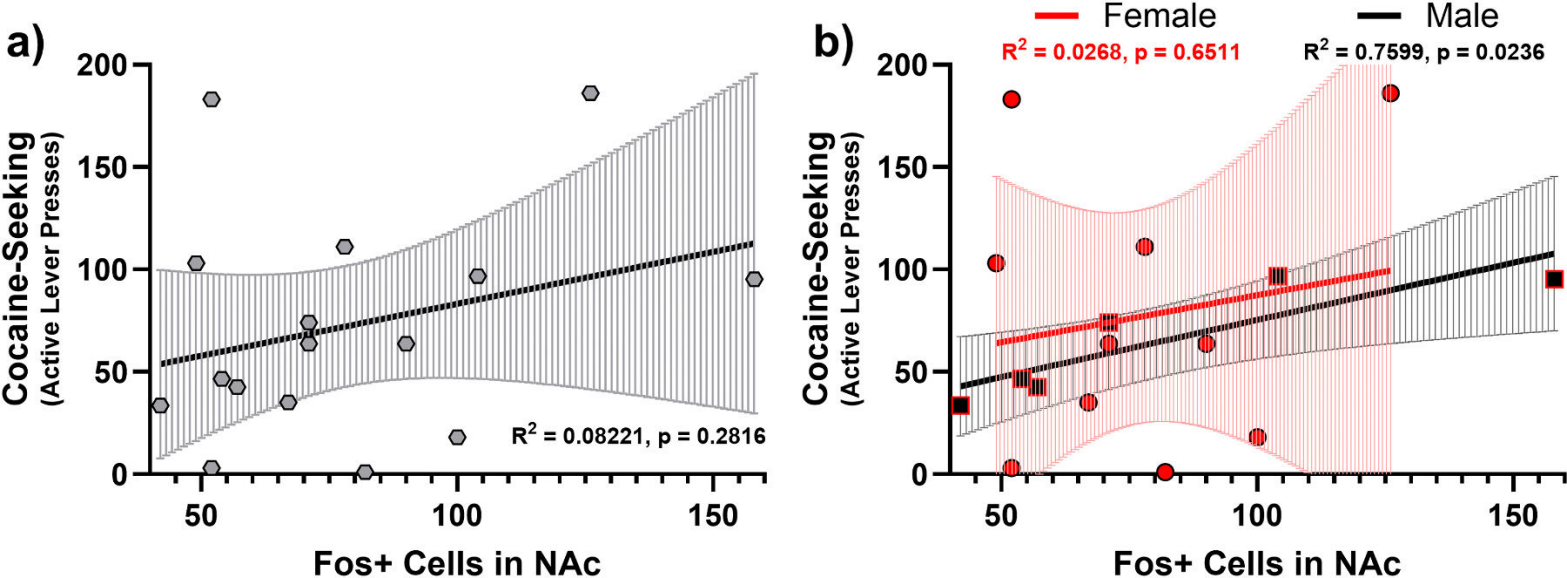
Sex-dependent relationship between NAc Fos expression and cocaine seeking. Error bars represent 95 % confidence intervals.

To determine whether any association between NAc Fos expression and cocaine seeking existed, we conducted regression and correlation analyses on NAc Fos and cocaine-seeking behavior. When combining female and male subjects, cocaine seeking (active lever presses) was not significantly correlated with NAc Fos expression (R^2^ = 0.0822, p = 0.2816; Fig. 10a). However, males alone showed a significant positive correlation (R^2^ = 0.7599, p = 0.0236), while females alone showed no significant relationship (R^2^ = 0.0268, p = 0.6511; Fig. 10b).

## 4. Discussion

This study investigated NAc Fos expression related to cocaine-induced LMA (Experiment 1) and cocaine seeking following forced abstinence (Experiment 2) to identify whether NAc Fos expression was related to these behaviors and whether sex differences existed in the behaviors themselves, the underlying NAc Fos expression, or their relationship.

Use of the behavioral sensitization model in Experiment 1 revealed behavioral, but not Fos-based, sex differences. The cocaine dose (10 mg/kg/day for 10 days) did not induce behavioral sensitization in either sex but caused hyperlocomotion in females. Since previous studies reported behavioral sensitization with this (relatively low) dose [7,31,32], it was unexpected that behavioral sensitization did not occur. One possible explanation may be insufficient habituation to the locomotor chambers causing novelty-based LMA in saline-treated animals that weakened differences between treatment groups, or driving.stereotypy behaviors (e.g., rearing) rather than LMA, which may have limited LMA behavioral sensitization.However, these results do replicate the finding that females show greater locomotor response to cocaine than males [20,31].

NAc Fos expression did not differ between groups in Experiment 1. We found no relationship between NAc Fos and LMA in the saline-saline, saline-cocaine, or cocaine-saline groups, with only a trending (*p* < 0.10) relationship in the cocaine-cocaine group. Many behavioral sensitization studies use higher doses [6,8], and it is likely that a higher dose would have induced behavioral sensitization herein. However, it remains unclear whether a relationship between cocaine-induced behavioral sensitization and Fos expression in the NAc would emerge at a higher dose, or whether another factor (e.g., the role of learning) is necessary for that relationship. Context-induced sensitization, where cocaine is repeatedly paired with environmental cues, sensitizes NAc Fos expression [6,8], and cocaine induces greater NAc Fos expression when administered in a novel environment compared to the home cage [33]. Further studies are needed to clarify the role of NAc Fos in cocaine hyperactivity and that of cocaine-induced learning.

Experiment 2 confirmed that female rats self-administer more cocaine-infusions and exhibit greater cocaine-primed seeking behavior than males [34–36]. These results reinforce the well-supported hypothesis (discussed below) that biological sex impacts behavior related to reward and motivation, including drug-taking and drug-seeking.

Previous research links Fos expression to cue-induced cocaine seeking [10,11]. In agreement, we observed greater Fos expression in the NAc compared to the pSSC control region. While cue-induced and priming-induced cocaine seeking recruit unique circuits to drive cocaine seeking [3,37], these two circuits both involve the NAc, and our data support the involvement of NAc Fos expression specifically. Debate exists over whether the NAc core and/or shell drives cocaine seeking, as some studies report that only one of the two subregions contribute to relapse-like behavior [10,11,38]. However, we found no Fos expression differences between the subregions, suggesting both contribute.

We hypothesized that sex differences in NAc Fos expression would underlie the anticipated behavioral cocaine seeking sex effect. However, females and males did not differ in the number of Fos+ cells in the NAc following cocaine-primed seeking. Sex differences in NAc Fos expression following cue-induced cocaine-reinstatement have been reported in some regions (e.g., NAc shell) [23], but we did not replicate this effect following cocaine-priming. This may be attributable to the fact that cued and primed seeking recruit slightly different neurocircuitry [37,38] or to other methodological differences between studies.

Additionally, we expected NAc Fos expression to correlate with cocaine seeking in both sexes. Interestingly, we observed this correlation only in males. Prior findings are mixed: Zhou et al. found a correlation between cue-induced cocaine reinstatement and NAc shell Fos expression in both sexes [23], though they did not report sex-specific analyses for this relationship. However, Kufahl et al. report no relationship between NAc Fos expression and cue-induced cocaine seeking in male rats [39]. Causal experiments to test the role of NAc Fos expression in cocaine seeking in both sexes are necessary to address this remaining gap. It is possible, given the influence of gonadal hormones on motivated behaviors like cocaine-seeking [12,13,40], that estrous cycle variation in females modulated cocaine-seeking behavior and/or Fos expression, thereby weakening the relationship.

Taken together, Experiments 1 and 2 suggest that the number of Fos+ cells in the NAc does not drive sex differences in behavior. Organizational and activational sex differences influence behavioral sensitization, self-administration, and cocaine seeking [13,40], potentially affecting Fos+ cell properties beyond their number. For example, females with intact gonadal hormones show greater cocaine-induced behavioral sensitization than ovariectomized females or males [31], female rats self-administer more cocaine in high-estradiol phases of the estrous cycle [15], and estradiol treatment enhances cocaine self-administration [13,40] and reinstatement [34]. To date, limited literature has examined the influence of gonadal hormones on Fos expression. For example, estradiol administration induces hippocampal Fos expression in ovariectomized female rats [41]. High-estradiol female rats show greater cocaine-induced behavioral sensitization and resulting Fos immunoreactivity in the medial preoptic area (mPOA) of the hypothalamus than low-estradiol females [42]. Finally, female rats treated with estradiol in the mPOA performed better in cocaine conditioned place preference tasks and showed greater NAc Fos activation compared to controls [43].

In this study, we found a sex-dependent relationship between NAc Fos expression and cocaine seeking (Experiment 2), but not cocaine-induced behavioral sensitization (Experiment 1). Self-administration and seeking involved operant learning, while sensitization did not, suggesting Fos expression may be learning dependent. Indeed, much of the work examining Fos expression in the context of drug use relies on conditioned behaviors (reviewed in *28*), and future work should clarify whether Fos expression is associated only with learned behaviors.

Several limitations exist in the present study. One such consideration involves the use of different strains in Experiments 1 (Sprague-Dawley) and 2 (Wistar). These strains performed comparably on conditioned place preference [44] and on locomotor response to psychostimulant methylphenidate [45], suggesting that strain yielded minimal influence on the present studies. In Experiment 1, the cocaine dose was too low to elicit behavioral sensitization. This dose has produced behavioral sensitization in multiple studies [7,31,32], but the lack of behavior herein limits interpretation of the Fos data. It therefore remains unclear whether the lack of a relationship between locomotion and NAc Fos reflects a limited behavioral response or would arise with a higher dose. Additionally, the present work has yet to explore causally the role of NAc Fos expression in these behaviors. This study would have also benefitted from larger sample sizes and additional controls, such as a yoked-saline or novel context group, to clarify whether Fos expression was specific to cocaine-seeking behavior in Experiment 2. Finally, our results suggest that NAc Fos-expression is correlated with cocaine-primed seeking in males, but that other factors [7] (e.g., estrous phase) may influence the higher degree of cocaine seeking in females. We did not, however, explore other possible factors that may contribute to the observed behavioral sex difference in either experiment.

Sex-dependent factors observed in females may modify cocaine seeking beyond Fos expression, leading to increased variability in females in cocaine seeking and diminishing the correlational relationship between Fos expression and cocaine-seeking behavior that we observed in Experiment 2. Indeed, estradiol regulates dopamine dynamics that affect cocaine behaviors [46,47]. Additionally, cell-type specifics, proteomic, electrophysiological, and structural properties of reward circuitry differ between sexes, and these differences have been implicated in cocaine use. For example, Lopez et al. report sex differences in the proteome of the NAc at baseline [48]. Cocaine-induced dopamine release and reuptake is greater in females than males [40], and sex differences in dopaminergic cell presence in reward neurocircuitry have been reported [49]. The impacts of circulating sex hormones in females and organizational sex differences on cocaine behaviors have been examined, but whether these mechanisms regulate NAc Fos expression involved in these behaviors specifically, remains unknown, and future work should examine the intersection of known sex differences with the Fos+ cells that govern related cocaine taking and seeking behavior.

## References

[1] Kampman KM, The treatment of cocaine use disorder, Sci. Adv. 5 (2019) eaax1532. 31663022 10.1126/sciadv.aax1532 PMC6795516

[2] Taylor SB, Lewis CR, Olive MF, The neurocircuitry of illicit psychostimulant addiction: acute and chronic effects in humans, Subst. Abus. Rehabil. 4 (2013) 29–43. 10.2147/SAR.S39684 PMC3931688 24648786

[3] Cooper S, Robison AJ, Mazei-Robison MS, Reward circuitry in addiction, Neurotherapeutics 14 (2017) 687–697. 28324454 10.1007/s13311-017-0525-z PMC5509624

[4] Floresco SB, The nucleus accumbens: an interface between cognition, emotion, and action, Annu Rev. Psychol. 66 (2015) 25–52. 25251489 10.1146/annurev-psych-010213-115159

[5] Hans JPJ, Crombag S, Kristina Redmond, Robinson Terry E, Bruce T, Hope Locomotor sensitization to cocaine is associated with increased Fos expression in the accumbens, but Not. caudate (2002). 10.1016/s0166-4328(02)00196-1 12429408

[6] Mattson BJ, , Context-specific sensitization of cocaine-induced locomotor activity and associated neuronal ensembles in rat nucleus accumbens, Eur. J. Neurosci. 27 (2008) 202–212. 18093170 10.1111/j.1460-9568.2007.05984.x

[7] Santos-Baldaia RD, , Distinctive neuroanatomic regions involved in cocaine-induced behavioral sensitization in mice, Biomedicines 11 (2023). 10.3390/biomedicines11020383 PMC9953661 36830920

[8] Hope BT, Simmons DE, Mitchell TB, Kreuter JD, Mattson BJ, Cocaine-induced locomotor activity and Fos expression in nucleus accumbens are sensitized for 6 months after repeated cocaine administration outside the home cage, Eur. J. Neurosci. 24 (2006) 867–875. 16930414 10.1111/j.1460-9568.2006.04969.x

[9] Zhang J, , c-Fos facilitates the acquisition and extinction of cocaine-induced persistent changes, J. Neurosci. 26 (2006) 13287–13296. 17182779 10.1523/JNEUROSCI.3795-06.2006 PMC6675013

[10] Bobadilla AC, , Cocaine and sucrose rewards recruit different seeking ensembles in the nucleus accumbens core, Mol. Psychiatry 25 (2020) 3150–3163. 32985600 10.1038/s41380-020-00888-z PMC8532193

[11] Cruz FC, , Role of nucleus accumbens shell neuronal ensembles in context-induced reinstatement of cocaine-seeking, J. Neurosci. 34 (2014) 7437–7446. 24872549 10.1523/JNEUROSCI.0238-14.2014 PMC4035511

[12] Becker JB, Hu M, Sex differences in drug abuse, Front. Neuroendocr. 29 (2008) 36–47. 10.1016/j.yfrne.2007.07.003 PMC2235192 17904621

[13] Becker JB, Koob GF, Sex differences in animal models: focus on addiction, Pharm. Rev. 68 (2016) 242–263. 26772794 10.1124/pr.115.011163 PMC4813426

[14] Dos Anjos Rosario B, , The influence of sex and reproductive cycle on cocaine-induced behavioral and neurobiological alterations: a review, Exp. Brain Res 240 (2022) 3107–3140. 36264315 10.1007/s00221-022-06479-4

[15] Kokane SS, Perrotti LI, Sex differences and the role of estradiol in mesolimbic reward circuits and vulnerability to cocaine and opiate addiction, Front. Behav. Neurosci. 14 (2020) 74. 32508605 10.3389/fnbeh.2020.00074 PMC7251038

[16] Nicolas C, , Sex differences in opioid and psychostimulant craving and relapse: a critical review, Pharm. Rev. 74 (2022) 119–140. 34987089 10.1124/pharmrev.121.000367 PMC11060335

[17] Robbins SJ, Ehrman RN, Childress AR, O’Brien CP, Comparing levels of cocaine cue reactivity in male and female outpatients, Drug Alcohol Depend. 53 (1999) 223–230. 10080048 10.1016/s0376-8716(98)00135-5

[18] Elman I, Karlsgodt K, Gastfriend D, Gender differences in cocaine craving among non-treatment-seeking individuals with cocaine dependence, Am. J. Drug Alcohol Abus. 27 (2001) 193–202. 10.1081/ada-100103705 11417935

[19] Kosten T, Gawin F, Kosten T, Rounsaville B, Gender Differences in Cocaine Use and Treatment Response. Journal of Substance Abuse Treatement 10, 63–66. 10.1016/0740-5472(93)90100-g 8450576

[20] van Haaren F, Meyer ME, Sex differences in locomotor activity after acute and chronic cocaine administration, Pharm. Biochem Behav. 39 (1991) 923–927. 10.1016/0091-3057(91)90054-6 1763112

[21] Jackson LR, Robinson TE, Becker JB, Sex differences and hormonal influences on acquisition of cocaine self-administration in rats, Neuropsychopharmacology 31 (2006) 129–138. 15920500 10.1038/sj.npp.1300778

[22] Fuchs RA, Evans KA, Mehta RH, Case JM, See RE, Influence of sex and estrous cyclicity on conditioned cue-induced reinstatement of cocaine-seeking behavior in rats, Psychopharmacology 179 (2005) 662–672. 15682307 10.1007/s00213-004-2080-7

[23] Zhou L, , Fos expression induced by cocaine-conditioned cues in male and female rats, Brain Struct. Funct. 219 (2014) 1831–1840. 23832598 10.1007/s00429-013-0605-8 PMC3877704

[24] Guide for the Care and Use of Laboratory Animals. The National Academies Collection: Reports funded by National Institutes of Health (Washington (DC), ed. 8th, 2011).

[25] Matchynski JI, , Direct measurement of neuronal ensemble activity using photoacoustic imaging in the stimulated Fos-LacZ transgenic rat brain: a proof-of-principle study, Photoacoustics 24 (2021) 100297. 34522608 10.1016/j.pacs.2021.100297 PMC8426561

[26] Eagle AL, , Single prolonged stress effects on sensitization to cocaine and cocaine self-administration in rats, Behav. Brain Res. 284 (2015) 218–224. 25712697 10.1016/j.bbr.2015.02.027 PMC5370568

[27] Perrine SA, , Low- and high-cocaine intake affects the spatial and temporal dynamics of class IIa HDAC expression-activity in the nucleus accumbens and hippocampus of male rats as measured by [18F]TFAHA PET/CT neuroimaging, Addict. Neurosci. 4 (2022). 10.1016/j.addicn.2022.100046 PMC9762729 36540409

[28] Cruz FC, Javier Rubio F, Hope BT, Using c-fos to study neuronal ensembles in corticostriatal circuitry of addiction, Brain Res. 1628 (2015) 157–173. 25446457 10.1016/j.brainres.2014.11.005 PMC4427550

[29] Paxinos G, Watson C, The Rat Brain in Stereotaxic Coordinates 7th *Edition*. (elsevier), pp. 456.

[30] McMullin SD, Shields GS, Slavich GM, Buchanan TW, Cumulative lifetime stress exposure predicts greater impulsivity and addictive behaviors, J. Health Psychol. 26 (2021) 2921–2936. 32643970 10.1177/1359105320937055 PMC7794090

[31] Hu M, Becker JB, Effects of sex and estrogen on behavioral sensitization to cocaine in rats, J. Neurosci. 23 (2003) 693–699. 12533629 10.1523/JNEUROSCI.23-02-00693.2003 PMC6741882

[32] Liu Y, Matsumoto RR, Alterations in fos-related antigen 2 and sigma1 receptor gene and protein expression are associated with the development of cocaine-induced behavioral sensitization: time course and regional distribution studies, J. Pharm. Exp. Ther. 327 (2008) 187–195. 10.1124/jpet.108.141051 18591217

[33] Uslaner J, , Environmental context modulates the ability of cocaine and amphetamine to induce c-fos mRNA expression in the neocortex, caudate nucleus, and nucleus accumbens, Brain Res. 920 (2001) 106–116. 11716816 10.1016/s0006-8993(01)03040-2

[34] Doncheck EM, , Estradiol regulation of the prelimbic cortex and the reinstatement of cocaine seeking in female rats, J. Neurosci. 41 (2021) 5303–5314. 33879537 10.1523/JNEUROSCI.3086-20.2021 PMC8211550

[35] Kippin TE, , Potentiation of cocaine-primed reinstatement of drug seeking in female rats during estrus, Psychopharmacology 182 (2005) 245–252. 16001116 10.1007/s00213-005-0071-y

[36] Lynch WJ, Carroll ME, Reinstatement of cocaine self-administration in rats: sex differences, Psychopharmacology 148 (1999) 196–200. 10.1007/s002130050042 10663435

[37] Farrell MR, Schoch H, Mahler SV, Modeling cocaine relapse in rodents: Behavioral considerations and circuit mechanisms, Prog. Neuropsychopharmacol. Biol. Psychiatry 87 (2018) 33–47. 29305936 10.1016/j.pnpbp.2018.01.002 PMC6034989

[38] Kalivas PW, McFarland K, Brain circuitry and the reinstatement of cocaine-seeking behavior, Psychopharmacology 168 (2003) 44–56. 12652346 10.1007/s00213-003-1393-2

[39] Kufahl PR, , c-Fos expression associated with reinstatement of cocaine-seeking behavior by response-contingent conditioned cues, Synapse 63 (2009) 823–835. 19533625 10.1002/syn.20666 PMC2748778

[40] Becker JB, Chartoff E, Sex differences in neural mechanisms mediating reward and addiction, Neuropsychopharmacology 44 (2019) 166–183. 29946108 10.1038/s41386-018-0125-6 PMC6235836

[41] Rudick CN, Woolley CS, Estradiol induces a phasic Fos response in the hippocampal CA1 and CA3 regions of adult female rats, Hippocampus 10 (2000) 274–283. 10902897 10.1002/1098-1063(2000)10:3<274::AID-HIPO8>3.0.CO;2-Q

[42] Martz JR, Vasquez A, Gillette R, Gore AC, Dominguez JM, The medial preoptic area and acute cocaine’s stimulant effects in rats: potential influences of estradiol and biological sex, Horm. Behav. 148 (2023) 105296. 36528006 10.1016/j.yhbeh.2022.105296 PMC9892259

[43] Robison CL, Martz JR, Dominguez JM, Influence of preoptic estradiol on behavioral and neural response to cocaine in female Sprague-Dawley rats, Psychopharmacology 235 (2018) 663–672. 29204804 10.1007/s00213-017-4800-9 PMC5823731

[44] Bardo MT, Rowlett JK, Harris MJ, Conditioned place preference using opiate and stimulant drugs: a meta-analysis, Neurosci. Biobehav. Rev. 19 (1995) 39–51. 7770196 10.1016/0149-7634(94)00021-r

[45] Yang PB, Amini B, Swann AC, Dafny N, Strain differences in the behavioral responses of male rats to chronically administered methylphenidate, Brain Res. 971 (2003) 139–152. 12706230 10.1016/s0006-8993(02)04240-3

[46] Calipari ES, , Dopaminergic dynamics underlying sex-specific cocaine reward, Nat. Commun. 8 (2017) 13877. 28072417 10.1038/ncomms13877 PMC5234081

[47] Shams WM, Sanio C, Quinlan MG, Brake WG, 17beta-Estradiol infusions into the dorsal striatum rapidly increase dorsal striatal dopamine release in vivo, Neuroscience 330 (2016) 162–170. 27256507 10.1016/j.neuroscience.2016.05.049

[48] Lopez AJ, , Cocaine self-administration induces sex-dependent protein expression in the nucleus accumbens, Commun. Biol. 4 (2021) 883. 34272455 10.1038/s42003-021-02358-w PMC8285523

[49] Kritzer MF, Creutz LM, Region and sex differences in constituent dopamine neurons and immunoreactivity for intracellular estrogen and androgen receptors in mesocortical projections in rats, J. Neurosci. 28 (2008) 9525–9535. 18799684 10.1523/JNEUROSCI.2637-08.2008 PMC2613180

